# Distinguishing Admissions Specifically for COVID-19 From Incidental SARS-CoV-2 Admissions: National Retrospective Electronic Health Record Study

**DOI:** 10.2196/37931

**Published:** 2022-05-18

**Authors:** Jeffrey G Klann, Zachary H Strasser, Meghan R Hutch, Chris J Kennedy, Jayson S Marwaha, Michele Morris, Malarkodi Jebathilagam Samayamuthu, Ashley C Pfaff, Hossein Estiri, Andrew M South, Griffin M Weber, William Yuan, Paul Avillach, Kavishwar B Wagholikar, Yuan Luo, The Consortium for Clinical Characterization of COVID-19 by EHR (4CE), Gilbert S Omenn, Shyam Visweswaran, John H Holmes, Zongqi Xia, Gabriel A Brat, Shawn N Murphy

**Affiliations:** 1 Laboratory of Computer Science Department of Medicine Massachusetts General Hospital Boston, MA United States; 2 Department of Preventive Medicine Northwestern University Chicago, IL United States; 3 Center for Precision Psychiatry Massachusetts General Hospital Boston, MA United States; 4 Department of Surgery Beth Israel Deaconess Medical Center Harvard Medical School Boston, MA United States; 5 Department of Biomedical Informatics University of Pittsburgh Pittsburgh, PA United States; 6 Section of Nephrology Department of Pediatrics Brenner Children's, Wake Forest School of Medicine Winston Salem, NC United States; 7 see Acknowledgments; 8 Center for Computational Medicine & Bioinformatics University of Michigan Ann Arbor, MI United States; 9 Department of Biostatistics, Epidemiology, and Informatics Perelman School of Medicine University of Pennsylvania Philadelphia, PA United States; 10 Department of Neurology University of Pittsburgh Pittsburgh, PA United States; 11 Department of Neurology Massachusetts General Hospital Boston, MA United States

**Keywords:** COVID-19, medical informatics, public health, phenotype, electronic health records, clinical research informatics, health data, SARS-CoV-2, patient data, health care

## Abstract

**Background:**

Admissions are generally classified as COVID-19 hospitalizations if the patient has a positive SARS-CoV-2 polymerase chain reaction (PCR) test. However, because 35% of SARS-CoV-2 infections are asymptomatic, patients admitted for unrelated indications with an incidentally positive test could be misclassified as a COVID-19 hospitalization. Electronic health record (EHR)–based studies have been unable to distinguish between a hospitalization specifically for COVID-19 versus an incidental SARS-CoV-2 hospitalization. Although the need to improve classification of COVID-19 versus incidental SARS-CoV-2 is well understood, the magnitude of the problems has only been characterized in small, single-center studies. Furthermore, there have been no peer-reviewed studies evaluating methods for improving classification.

**Objective:**

The aims of this study are to, first, quantify the frequency of incidental hospitalizations over the first 15 months of the pandemic in multiple hospital systems in the United States and, second, to apply electronic phenotyping techniques to automatically improve COVID-19 hospitalization classification.

**Methods:**

From a retrospective EHR-based cohort in 4 US health care systems in Massachusetts, Pennsylvania, and Illinois, a random sample of 1123 SARS-CoV-2 PCR-positive patients hospitalized from March 2020 to August 2021 was manually chart-reviewed and classified as “admitted with COVID-19” (incidental) versus specifically admitted for COVID-19 (“for COVID-19”). EHR-based phenotyping was used to find feature sets to filter out incidental admissions.

**Results:**

EHR-based phenotyped feature sets filtered out incidental admissions, which occurred in an average of 26% of hospitalizations (although this varied widely over time, from 0% to 75%). The top site-specific feature sets had 79%-99% specificity with 62%-75% sensitivity, while the best-performing across-site feature sets had 71%-94% specificity with 69%-81% sensitivity.

**Conclusions:**

A large proportion of SARS-CoV-2 PCR-positive admissions were incidental. Straightforward EHR-based phenotypes differentiated admissions, which is important to assure accurate public health reporting and research.

## Introduction

Despite the ongoing COVID-19 pandemic and the dozens of research groups and consortia worldwide that continue to utilize clinical data available in electronic health records (EHRs), critical gaps remain in both our understanding of COVID-19 and how to accurately predict poor outcomes, including hospitalization and mortality [1-4].

One of the most prominent gaps in the field is how to distinguish hospital admissions specifically for COVID-19-related indications (eg, severe disease with respiratory failure) from an incidentally positive SARS-CoV-2 polymerase chain reaction (PCR) test in admissions for an unrelated reason (eg, a broken leg). Approximately 800,000 new SARS-CoV-2 cases are being reported daily, and approximately 150,000 patients are hospitalized with a positive SARS-CoV-2 PCR test [5]. Misclassification of incidental COVID-19 during hospitalizations is common [5] and raises research and public health concerns. For example, deleterious effects on health care system resource disbursement or utilization as well as on local and regional social and economic structure and function can result from inaccurate reporting of incidental cases of SARS-CoV-2.

Misclassification in research studies occurs because patients are usually considered COVID-19 patients if they have a recent positive SARS-CoV-2 PCR test or the *International Classification of Diseases, Tenth Revision* (ICD-10) diagnosis code U07, which, according to guidelines, is equivalent to a positive test [6]. This approach has been used in most COVID-19 studies published to date [7,8] and is in line with Centers of Disease Control and Prevention (CDC) guidelines, which treat positive SARS-CoV-2 PCR tests as confirmed cases [9]. Given that at least 35% of SARS-CoV-2 cases are asymptomatic, patients seeking unrelated care are erroneously classified as COVID hospitalizations [10-14]. The magnitude of this misclassification has increased over time as health care systems began to be less restrictive after the second wave and elective surgeries were again performed starting in the second quarter of 2021.

A potential solution is EHR-based phenotyping, which identifies patient populations of interest based on proxies derived from EHR observations. EHR phenotypes are developed by first performing manual chart review to classify cases and then applying a machine learning or statistical reasoning method to the EHR data to create an explainable predictive model [15,16]. For example, a phenotyping study of bipolar disorder found that true bipolar disorder is correlated with a set of several EHR features [17]. Our previous work validated a “severe COVID-19” phenotype in the Consortium for Clinical Characterization of COVID-19 by EHR (4CE) network using both chart review and comparison across sites [18,19]. 4CE is a diverse international network of over 300 hospitals engaged in collaborative COVID-19 research [2,20,21].

The Massachusetts Department of Public Health has recently begun using a simple phenotype to report COVID-19 hospitalizations [22,23]. Although it is based on treatment recommendations and not a gold standard, it illustrates the interest in EHR-based phenotyping for COVID-19.

In this study, we utilized EHR data from 60 hospitals across 4 US health care systems in 4CE, combined with clinical expertise, data analytics, and manual EHR chart review, to determine whether patients admitted to the hospital and who had a positive SARS-CoV-2 PCR test were hospitalized for COVID-19 (for-COVID-19 group) or were admitted for a different indication and simply had an incidental positive test (admitted-with-COVID-19 group).

## Methods

### Sites

We selected a sample of 4 4CE sites across the United States to participate in the development of our for-COVID-19 hospitalization phenotype. These sites included the Beth Israel Deaconess Medical Center (BIDMC), Mass General Brigham (MGB), Northwestern University (NWU), and the University of Pittsburgh/University of Pittsburgh Medical Center (UPITT). Each site involved at least 1 *clinical* expert (for chart review and manual annotation) and 1 *data analytics* expert (to apply various analytic filtering approaches). Eligible patients for this study were those included in the 4CE COVID-19 cohort: all hospitalized patients (pediatric and adult) with their first positive SARS-CoV-2 PCR test 7 days before to 14 days after hospitalization [2].

### Chart Review

Each development site randomly sampled an equal number of admissions in each quarter (BIDMC, MGB) or month (NWU, UPITT) from their cohort of SARS-CoV-2 PCR-positive patients over the period of March 2020 until at least March 2021 (N=1123). Clinical experts reviewed the charts in the EHRs and recorded whether these patients were admitted for COVID-19-related reasons, as defined later. The total number of chart reviews per site was somewhat variable and determined by availability of the clinical experts. Participating sites and the number of chart reviews are listed in Table 1.

To develop chart review criteria, a 4CE subgroup met during March-July 2021. The group consists of about 20 researchers in 4CE, with a mixture of physicians, medical informaticians, and data scientists. In the process, dozens of real patient charts were considered, and edge cases were discussed until consensus was reached on the minimal chart review necessary to determine the reason a patient was hospitalized.

**Figure 1:**
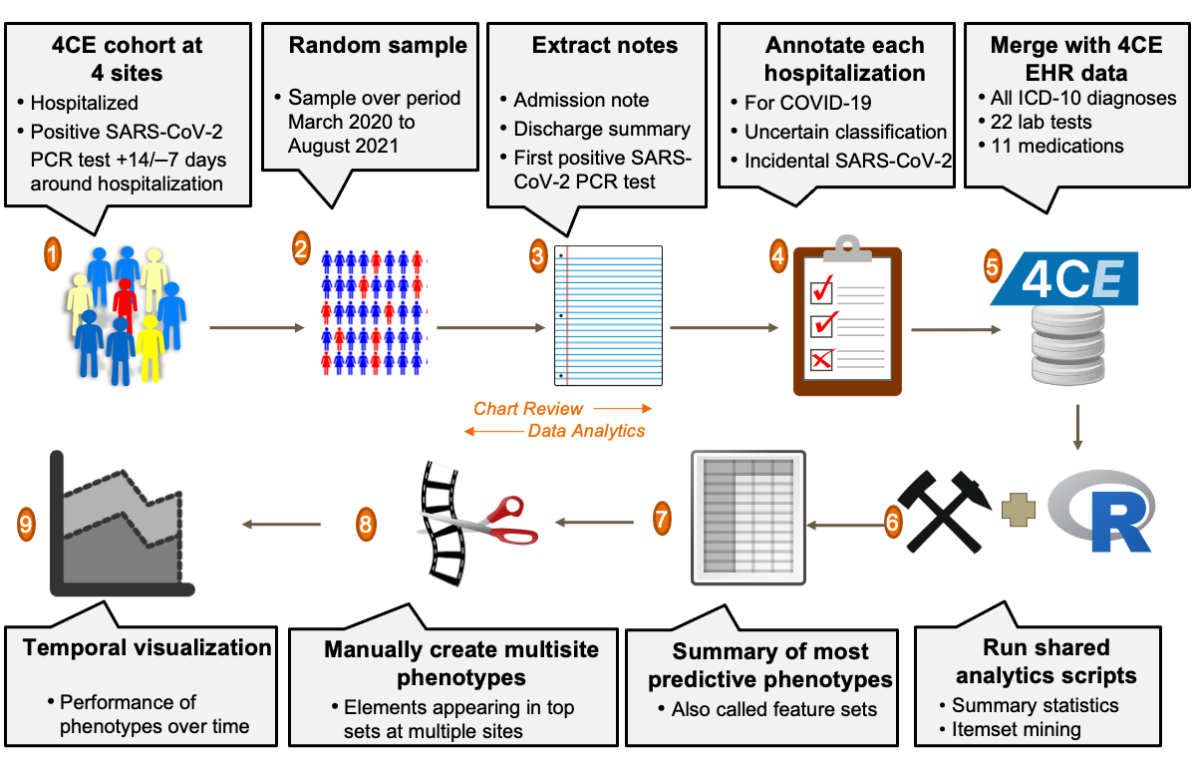
The chart review process. (1-2) At each site, an equal number of patients admitted with a positive SARS-CoV-2 PCR test were sampled by quarter or by month. (3-4) A chart reviewer at the site examined primarily the admission note, discharge summary (or death note), and laboratory values for the hospitalization to classify as admitted for COVID-19, incidental SARS-CoV2, or uncertain. (5-6) These classifications were then merged with 4CE EHR data for use with shared analytic scripts in R. (7-8) The top phenotypes at each site output by the data mining algorithm were summarized, and this was used to manually construct feature sets to be used across sites by selecting components that appeared in step 7 at multiple sites. (9) The performance over time of the top multisite phenotypes was visualized. 4CE: Consortium for Clinical Characterization of COVID-19 by EHR; EHR: electronic health record; ICD-10: International Classification of Diseases, Tenth Revision; PCR: polymerase chain reaction.

Based on the developed criteria, chart reviewers (1 per site, except at the BIDMC, where there were 2) classified the patients based on review of primarily the admission note, discharge summary (or death note), and laboratory values for the hospitalization. Each site had Institutional Review Board (IRB) approval to view the charts locally, and only deidentified aggregate summaries were presented to the subgroup. Each site summarized the chart reviews in a spreadsheet that was then linked to the site’s 4CE EHR data, wherein medical record numbers were replaced with 4CE’s patient pseudoidentifiers, and criteria classifications were coded as an integer. The 4CE EHR data set is a COVID-19-related subset of raw EHR data consisting of selected laboratory test, medication, and procedure categories and all available ICD-10 diagnosis codes. The data dictionary is explained in more detail in Multimedia Appendix 1. The chart review process is presented visually in steps 1-5 of Figure 1.

We developed an R script (R Core Team) at the MGB to perform basic data summarization. This did the following: calculated chart review summary statistics, aggregated data on ICD-10 diagnosis codes used during the hospitalization to compare to the chart review classification, and generated a bubble plot that visualizes the change in proportion of hospitalizations, specifically for COVID-19, among all chart reviews over the course of the pandemic, by month. A trendline was fitted with locally estimated scatterplot smoothing (loess) regression using ggplot2 and was weighted by the number of chart reviews performed that month. Each participating health care site ran the R script on its chart-reviewed patient cohort.

**Table 1:** Participating health care systems’ overall characteristics and the number and period of chart reviews performed for this study.

Participating site	Hospitals, n	Inpatient discharges per year, n	Number of chart reviews performed, n	Chart review time period, start date-end date
BIDMC^a^	1	40,752	400	March 2020-March 2021
MGB^b^	10	163,521	406	March 2020-July 2021
NWU^c^	10	103,279	70	March 2020-February 2021
UPITT^d^	39	369,300	247	April 2020-August 2021

^a^BIDMC: Beth Israel Deaconess Medical Center.

^b^MGB: Mass General Brigham.

^c^NWU: Northwestern University.

^d^UPITT: University of Pittsburgh/University of Pittsburgh Medical Center.

### Phenotypes Using Hospital System Dynamics Phenotyping

**Figure 2:**
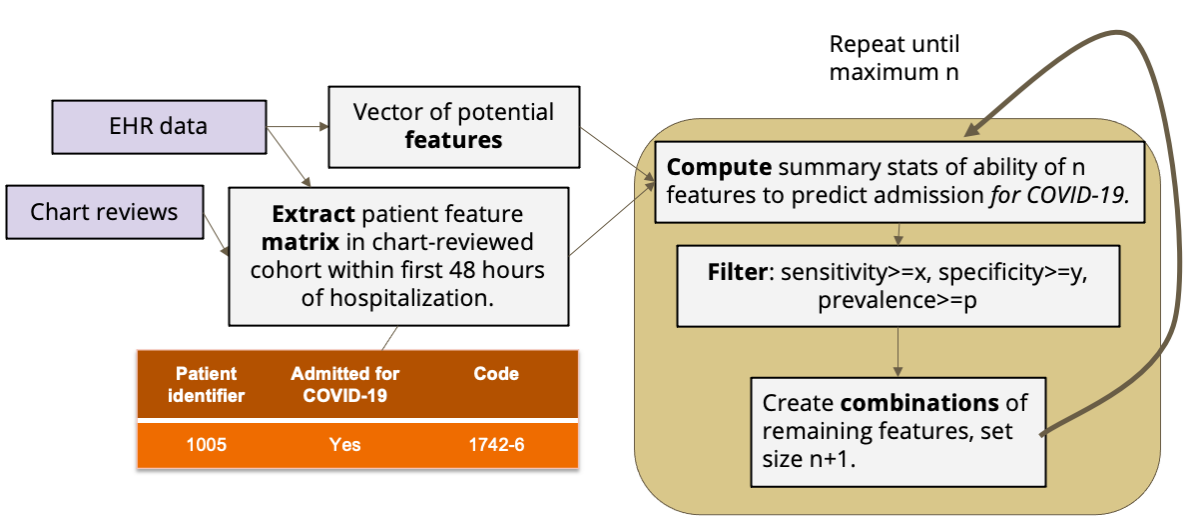
Design of the phenotyping algorithm. Predictive feature sets of iteratively larger size were selected based on their sensitivity and specificity in correctly identifying COVID-19-specific admissions using 4CE EHR data and chart reviews. We chose the following parameters after testing various thresholds at all 4 sites: AND feature sets, x=0.40, y=0.20, p=0.30; OR feature sets x=0.10, y=0.50, p=0.20; and single features: x=y=p=0. 4CE: Consortium for Clinical Characterization of COVID-19 by EHR; EHR: electronic health record.

We developed an algorithm as an R script to choose phenotypes of admissions specifically for COVID-19, using established hospital dynamics measures of ordering/charting patterns in the EHRs (eg, presence of laboratory tests rather than laboratory results) [16,24]. The algorithm uses a variation of the Apriori item set–mining algorithm [25,26]. Apriori, which has been utilized in other EHR studies, uses a hill-climbing approach to find iteratively larger item sets that meet some summary statistic constraint [27,28]. Apriori, like other market basket analyses, is advantageous when the labeled data are small, because it discovers statistical properties of the underlying data, rather than developing a separate predictive model that must be evaluated. Therefore, it does not require a data split between a training and a test set, which would further limit the sample size. The original algorithm chose rules that maximized the positive predictive value (PPV) and had at least a minimum prevalence in the data set. More recent variants use other summary statistics [29] because the PPV, which measures the likelihood a positive is a true positive, is highly affected by population prevalence (which shifts dramatically over time with COVID-19). Therefore, our algorithm used sensitivity and specificity. A visual representation of our algorithm is shown in Figure 2. Item sets of size 1 are chosen that meet certain minimum prediction thresholds, and then these are combined into item sets of size 2 and again filtered by the thresholds, and so forth up to a maximum item set size.

We applied our algorithm to find patterns in 4CE EHR data at each site using the presence of medications, laboratory tests, and diagnoses to select the best phenotypes. (Laboratory test results are included in the 4CE data set but were not included in this analysis, because it does not fit with the principles of hospital system dynamics [HSD].) We further compared the output at each site to see whether there were similarities (eg, transfer learning was applicable). We considered 2 cases: data that would be available in near-real time during a hospitalization (laboratory tests) and data that would be available for a retrospective analysis (including laboratory and medication facts and diagnosis codes, which are usually not coded until after discharge).

Sites exported phenotypes with sensitivity of at least 0.60, ordered by specificity in descending order. (Site B applied a slightly lower sensitivity threshold because no phenotypes with sensitivity of at least 0.60 were available.) Specificity was chosen as the sorting variable because it measures the phenotype’s ability to detect and remove incidental SARS-CoV-2 admissions—a good measure of overall performance. Sensitivity, in contrast, measures the ability to select for-COVID-19 admissions, which can be easily maximized by simply selecting all patients. Groups of phenotypes were manually summarized into conjunctive normal form by combining AND and OR phenotypes at each site, when possible, and reporting a sensitivity and specificity range for the final combined phenotype. We excluded feature sets that were more complex but with the same performance as a simpler feature set.

We also ran our phenotyping program to find the most predictive individual features at each site during every 6-month period of the pandemic, beginning January 2020. This analysis allowed us to examine the trend of HSD as the pandemic progressed.

The final piece of analysis involved selecting multisite phenotypes and plotting their performance over time. First, we selected the features that appeared at multiple sites from the best phenotypes at each site. We used these to manually construct multisite phenotypes. We optimized these using MGB data by manually adding/removing OR components based on performance, because adding too many OR components degrades the specificity. We ran these constructed phenotypes at each site to ascertain their performance characteristics.

This data-mining process can be seen visually in Figure 1, steps 6-8.

### Temporal Visualization of Phenotypes

We also developed a temporal visualization used at each site (step 9 of Figure 1). The visualization shows 3 lines: a solid line showing the total number of patients in the site’s 4CE cohort (ie, admitted with a positive SARS-CoV-2 PCR test), a dashed line showing the total number of those patients after filtering to select patients admitted specifically for COVID-19 (ie, removing all patients who do not meet the phenotyping feature set criteria), and a dotted line showing the difference between the solid line and the dashed line (ie, patients removed from the cohort in the dashed line). Dots on the graph visualize the performance on the chart-reviewed cohort. Green dots on each line show patients who were correctly classified by the phenotype, according to the chart review. Likewise, orange dots on each line show incorrect classifications. Dot size is proportional to the number of chart reviews.

Importantly, all review and analysis were performed by local experts at each site, and only the final aggregated results were submitted to a central location for finalization. This approach is a hallmark of 4CE—keeping data close to local experts and only sharing aggregated results. It reduces regulatory complexity around data sharing and keeps those who know the data best involved in the analysis.

All our software tools were implemented as R programs. They were developed at the MGB and tested by all 4 sites. The code is available as open source [30].

### Ethical Considerations

IRB approval was obtained at the BIDMC (#2020P000565), the MGB (#2020P001483), the UPITT (STUDY20070095), and the NWU (STU00212845). Participant informed consent was waived by each IRB because the study involved only retrospective data and no individually identifiable data were share outside of each site’s local study team. Site names were anonymized (to sites A, B, C, and D) to comply with hospital privacy policies. At the MGB and the BIDMC, any counts of patients were blurred with a random number +/–3 before being shared centrally. Our previous work shows that for large counts, pooling blurred counts has minimal impact on the overall accuracy of the statistics [31]. At all sites, any counts <3 were censored. All other statistics (eg, percentages, differences, CIs, *P* values) were preserved.

## Results

### Chart Review

**Figure 3:**
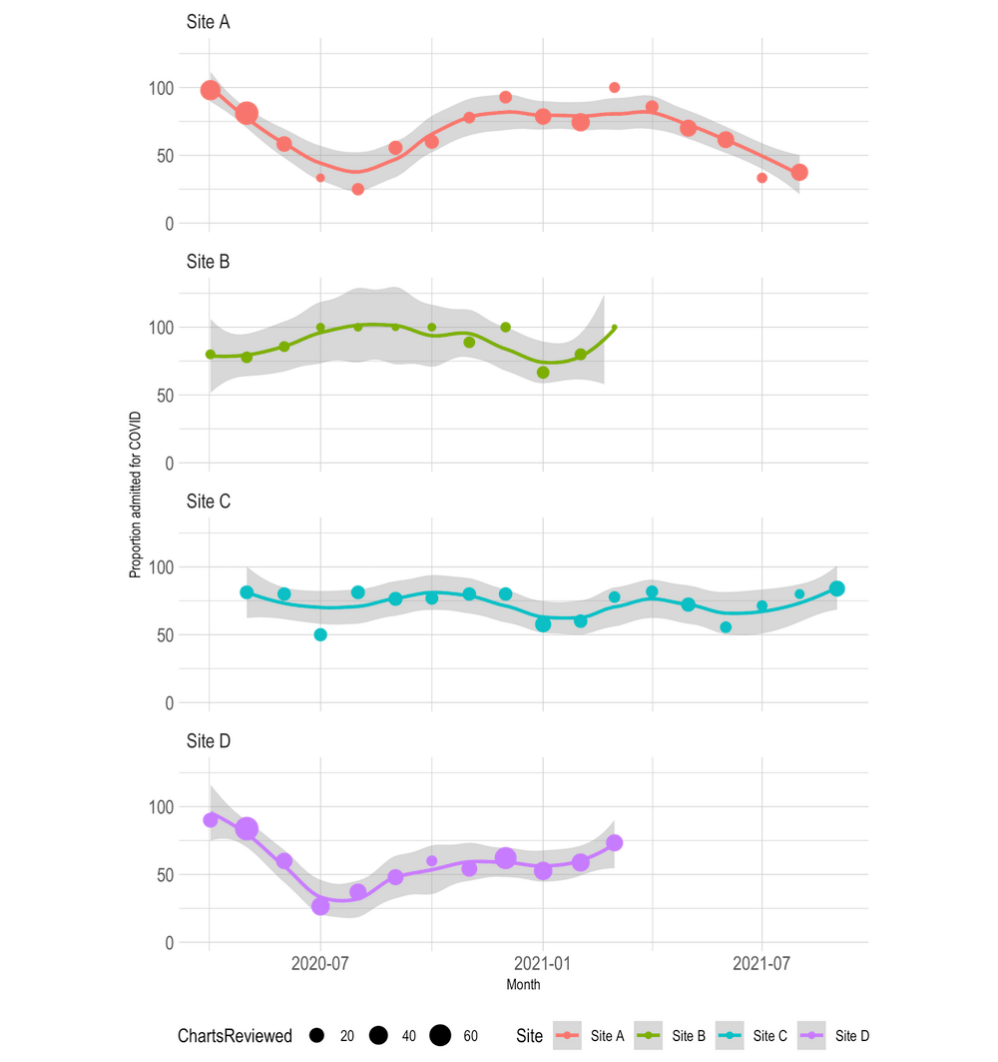
Chart-reviewed proportion of admissions specifically for COVID-19 among all chart reviews by month at each site. The bubble size shows the relative number of patient chart reviews performed that month. The trendline was weighted by bubble size and was performed using locally weighted least squares (loess) regression. Note that the y axis and 95% CI limits extend above 100%.

The final chart review criteria are shown in Table 2. (See the Methods section for details.) Across the 4 sites, 764 (68%) of 1123 patients were admitted *for* COVID-19, 292 (26%) patients were admitted with *incidental* SARS-CoV-2, and 67 (6%) were uncertain (Table 3). The 4 sites included the BIDMC, the MGB, the UPITT, and the NWU. A site-by-site breakdown, both overall and by individual criteria, is also shown in Table 3. A demographic characterization of the chart-reviewed cohort at each site is shown in Table 4. Plots of the proportion of hospitalizations specifically for COVID-19 among all chart reviews by month over the course of the pandemic are shown in Figure 3. Finally, Tables 5 and 6 show the top 10 ICD-10 diagnoses that were assigned to patients with a date in the first 48 hours after admission in *for-*COVID-19 versus *incidental-*COVID-19 groups. In all results, each site is labeled with a random but consistent letter (A, B, C, or D) to comply with hospital privacy policies.

**Table 2:** Summary of the chart review criteria developed by the 4CE^a^ subgroup of physicians, medical informaticians, and data scientists.

Chart-reviewed classification	Criteria
Admitted specifically *for* COVID-19	Symptoms on admission *were attributable* to COVID-19, and clinicians admitted patients for COVID-19-related care. The symptoms included: Respiratory insufficiency Blood clots in vital organs Hemodynamic changes Other common viral symptoms, such as cough and fever Admitted for non-COVID-19 issue but developed any of the above symptoms while hospitalized
Admitted incidentally *with* COVID-19	The admission history was *unlikely* to be related to COVID-19, and clinicians did not specifically admit the patient for COVID-19-related care. This admission could be due to: Trauma Procedure or operation requiring hospitalization Term labor Alternative causes, including drug overdose, cancer progression, and nonrespiratory severe infection
Uncertain	Symptoms on admission *may have been* related to COVID-19, and clinicians considered COVID-19 exacerbation during hospitalization. The symptoms included: Preterm labor Liver dysfunction Graft failure Immune system dysfunction Alternative causes, including sickle cell crisis, failure to thrive, and altered mental status

^a^4CE: Consortium for Clinical Characterization of COVID-19 by EHR^b^.

^b^EHR: electronic health record.

**Table 3:** Proportion of chart-reviewed patients admitted specifically for COVID-19 vs admitted with incidental SARS-CoV-2, overall and stratified by site, with a detailed criteria breakdown. A detailed breakdown at site D could not be included, because their process did not record the specific criteria for each classification. Note that cells with 0% are still included to show all the chart review criteria.

Category		Site A (N=406), n (%)	Site B (N=70), n (%)	Site C (N=247), n (%)	Site D (N=400), n (%)	Overall (N=1123), n (%)
**Admitted specifically *for* COVID-19**						764 (68)
	All	288 (71)	59 (84)	180 (73)	240 (60)	N/A^a^
	Respiratory insufficiency	202 (50)	36 (51)	128 (52)	N/A	N/A
	Blood clot	6 (1)	<3 (<5)	<3 (<5)	N/A	N/A
	Hemodynamic changes	<3 (<5)	<3 (<5)	<3 (<5)	N/A	N/A
	Other symptomatic COVID-19	71 (18)	19 (27)	47 (20)	N/A	N/A
	Not admitted for COVID-19 but developed 1 of the above criteria	8 (2)	<3 (<5)	5 (2)	N/A	N/A
**Admitted incidentally with COVID-19**						292 (26)
	All	85 (20)	9 (13)	54 (22)	144 (36)	N/A
	Full-term labor	18 (4)	<3 (<5)	<3 (<5)	N/A	N/A
	Procedure	8 (2)	<3 (<5)	9 (4)	N/A	N/A
	Trauma	<3 (<5)	<3 (<5)	<3 (<5)	N/A	N/A
	Other not COVID-19	50 (13)	6 (9)	44 (18)	N/A	N/A
**Uncertain**						67 (6)
	All	33 (8)	<3 (<5)	10 (4)	16 (4)	N/A
	Immune dysfunction	<3 (<5)	<3 (<5)	<3 (<5)	N/A	N/A
	Early labor	<3 (<5)	<3 (<5)	<3 (<5)	N/A	N/A
	Liver dysfunction	<3 (<5)	<3 (<5)	<3 (<5)	N/A	N/A
	Graft failure	<3 (<5)	<3 (<5)	<3 (<5)	N/A	N/A
	Other possible COVID-19	31 (8)	<3 (<5)	10 (4)	N/A	N/A

^a^N/A: not applicable.

**Table 4:** Demographic characterization of the chart-reviewed cohort by site. For each row, the count and percentage (in parentheses) at each site are shown. Two sites did not report Hispanic/Latino. N values for each site are shown in the header; these might not exactly match the summation of each category due to blurring requirements.

Category		Site A (N=406), n (%)	Site B (N=70), n (%)	Site C (N=247), n (%)	Site D (N=400), n (%)
**Age (years)**					
	0-25	14 (4)	11 (14)	4 (1)	11 (3)
	26-49	95 (23)	15 (21)	26 (10)	76 (18)
	50-69	138 (35)	22 (31)	99 (40)	135 (33)
	70-79	72 (17)	9 (13)	59 (24)	90 (22)
	80+	83 (20)	13 (18)	59 (24)	81 (19)
**Race**					
	Asian	8 (2)	2 (3)	5 (2)	17 (4)
	Black	60 (14)	9 (13)	58 (23)	97 (24)
	Hispanic/Latino	21 (6)	N/A^a^	N/A	55 (14)
	White	78 (19)	50 (71)	179 (72)	173 (42)
	No information	230 (58)	8 (11)	5 (2)	61 (14)
**Sex**					
	Male	200 (50)	42 (60)	121 (49)	188 (47)
	Female	200 (50)	28 (40)	126 (51)	211 (52)

^a^N/A: not applicable.

**Table 5:** Top 10 ICD-10^a^ diagnoses among patients’ charts reviewed as admitted specifically for COVID-19, with the proportion of patients with each diagnosis at each site. Each patient might have multiple diagnoses, and therefore, the sum might be greater than 100%.

ICD-10 diagnosis	Site A (N=288), n (%)	Site B (N=59), n (%)	Site C (N=180), n (%)	Site D (N=240), n (%)
U07.1 Covid-19	265 (92)	54 (92)	145 (80)	226 (95)
J12.89 Other Viral Pneumonia	125 (44)	24 (41)	64 (35)	173 (70)
I10 Essential (Primary) Hypertension	113 (39)	16 (27)	74 (41)	89 (37)
J96.01 Acute Respiratory Failure With Hypoxia	75 (26)	20 (34)	56 (31)	139 (58)
E78.5 Hyperlipidemia, Unspecified	79 (28)	4 (7)	69 (38)	108 (46)
N17.9 Acute Kidney Failure, Unspecified	74 (25)	4 (7)	40 (22)	94 (39)
K21.9 Gastro-Esophageal Reflux Disease Without Esophagitis	64 (22)	<3 (<3)	57 (31)	65 (26)
Z87.891 Personal History of Nicotine Dependence	56 (18)	<3 (<3)	44 (24)	66 (27)
R09.02 Hypoxemia	81 (29)	15 (25)	21 (12)	43 (17)
J12.82 Pneumonia due to COVID-19	72 (25)	12 (20)	39 (22)	35 (15)

^a^ICD-10: International Classification of Diseases, Tenth Revision.

**Table 6:** Top 10 ICD-10^a^ diagnoses among patients’ charts reviewed as admitted with incidental COVID-19, with the proportion of patients with each diagnosis at each site. Each patient might have multiple diagnoses, and therefore, the sum might be greater than 100%.

ICD-10 diagnosis	Site A (N=85), n (%)	Site B (N=9), n (%)	Site C (N=54), n (%)	Site D (N=144), n (%)
U07.1 Covid-19	63 (74)	5 (56)	40 (73)	122 (85)
N17.9 Acute Kidney Failure, Unspecified	12 (14)	<3 (<11)	12 (22)	24 (17)
E11.22 Type 2 Diabetes Mellitus with Diabetic Chronic Kidney Disease	5 (6)	<3 (<11)	7 (13)	23 (15)
E11.9 Type 2 Diabetes Mellitus Without Complications	12 (11)	<3 (<11)	4 (7)	14 (11)
D64.9 Anemia, Unspecified	13 (19)	<3 (<11)	5 (9)	10 (6)
E87.2 Acidosis	8 (6)	<3 (<11)	<3 (<5)	12 (10)
J12.89 Other Viral Pneumonia	<3 (<2)	<3 (<11)	4 (7)	15 (12)
J96.01 Acute Respiratory Failure With Hypoxia	6 (8)	<3 (<11)	4 (7)	13 (8)
D69.6 Thrombocytopenia, Unspecified	5 (7)	<3 (<11)	6 (11)	12 (7)
N18.6 End-Stage Renal Disease	6 (7)	<3 (<11)	5 (9)	6 (5)

^a^ICD-10: *International Classification of Diseases, Tenth Revision*.

### Phenotypes Using Hospital System Dynamics

Each site ran our HSD program to choose phenotypes of patients admitted *for COVID-19* versus patients admitted *incidentally with COVID-19*. The input of the program includes the chart-reviewed classifications and patient-level EHR data on the presence of 22 selected laboratory test types, 11 selected medication categories, 12 procedure categories, and all ICD-10 diagnosis codes that are dated within 48 hours of admission. This resulted in 1880 distinct features across all sites. (See Multimedia Appendix 1 for more information on the data dictionary.) The program selected 135 feature sets across all sites using these features. These were manually reduced to 32 (23.7%) by selecting the most predictive and removing duplicates and near-duplicates. These are summarized in Table 7, divided into phenotypes that use data that could be available immediately (“real time”) and phenotypes using all data available after discharge (“retrospective”). We also reported the prevalence at each site among all SARS-CoV-2 PCR-positive hospitalizations (not just among chart-reviewed patients), which is the proportion of patients meeting the criteria of the feature sets.

**Table 7:** Top phenotyping feature sets by specificity, with a sensitivity of at least 0.60 for detecting admissions specifically for COVID-19. The table is grouped into feature sets involving potentially real-time data (laboratory tests) and all available data (presence of laboratory tests, medications, and diagnosis codes). Note that laboratory test results are not included in the feature sets. Ranges are shown in the summary statistics because multiple rules with similar performance were summarized using conjunctive normal form.

Phenotyping feature set		Site	Sensitivity	Specificity	Prevalence (%)
“**Real-time” phenotypes (laboratory tests only)**					
	CRP^a^ AND (Total Bilirubin OR Ferritin OR LDH^b^) AND (Lymphocyte Count OR Neutrophil Count) AND Cardiac Troponin	D	0.65-0.72	0.85	67-71
	Ferritin AND LDH AND Cardiac Troponin AND (INR^c^ OR PTT^d^ OR Lymphocyte Count OR Neutrophil Count)	D	0.62-0.69	0.85	67-71
	CRP AND (LDH AND/OR Ferritin) AND Cardiac Troponin	A	0.67-0.70	0.89-0.90	72-77
	Procalcitonin OR D-dimer OR CRP OR Cardiac Troponin OR Ferritin	A	0.63-0.87	0.73-0.85	65-85
	Any 2 of: Procalcitonin, LDH, CRP	B	0.56-0.58	0.67	63-67
	D-dimer OR Ferritin OR CRP	C	0.26-0.37	0.86-0.93	54-58
“**Retrospective” phenotypes (laboratory tests, medications, and diagnosis codes)**					
	Total bilirubin AND (Ferritin OR LDH OR Lymphocyte Count OR Neutrophil Count) AND diagnosis of Other Viral Pneumonia (J12.89)	D	0.62-0.64	0.92	46-48
	Diagnosis of: Other Viral Pneumonia (J12.89) OR Acute Respiratory Failure with Hypoxia (J96.01) OR Anemia (D64.9)	D	0.70-0.74	0.82-0.88	50-63
	Diagnosis of: Other Viral Pneumonia (J12.89) OR Supplemental Oxygen (severe)	D	0.75	0.82	61
	CRP AND (LDH OR Ferritin) AND Cardiac Troponin	A	0.70	0.89	74-77
	Remdesivir OR Procalcitonin OR Other Viral Pneumonia (J12.89) OR Nonspecific Abnormal Lung Finding (R91.8) OR Shortness of Breath (R06.02) OR Other COVID Disease (J12.82)	A	0.68-0.72	0.85-0.95	58-74
	Hypoxemia (R09.02) OR Other Coronavirus as Cause of Disease (B97.29) OR Shortness of Breath (R06.02) OR Pneumonia (unspecified organism) (J18.9) OR Acute Respiratory Failure with Hypoxia (J96.01) OR Nonspecific Abnormal Lung Finding (R91.8)	B	0.63-0.68	0.89-0.99	54-67
	D-dimer OR ferritin OR CRP OR Other Viral Pneumonia (J12.89) OR Acute Respiratory Failure with Hypoxia (J96.01)	C	0.71-0.75	0.79-0.86	52-58

^a^CRP: C-reactive protein.

^b^LDH: lactate dehydrogenase.

^c^INR: international normalized ratio.

^d^PTT: partial thromboplastin time.

We examined the top individual features over time at all sites. In the first half of 2020, a diagnosis of “Other Viral Pneumonia” (J12.89) was the only strong predictor of an admission specifically for COVID-19 across all 4 sites. In the second half of 2020, the phenotyping algorithm began selecting laboratory tests, including C-reactive protein (CRP), troponin, ferritin, and lactate dehydrogenase (LDH). In addition, the diagnosis “Other Coronavirus as Cause of Disease” (B97.29) began to be used at site B. By 2021, remdesivir and the diagnosis “Pneumonia due to COVID-19” (J12.82) additionally came into widespread use and became predictive of admissions specifically for COVID at site A.

### Temporal Visualization of Phenotypes

**Figure 4:**
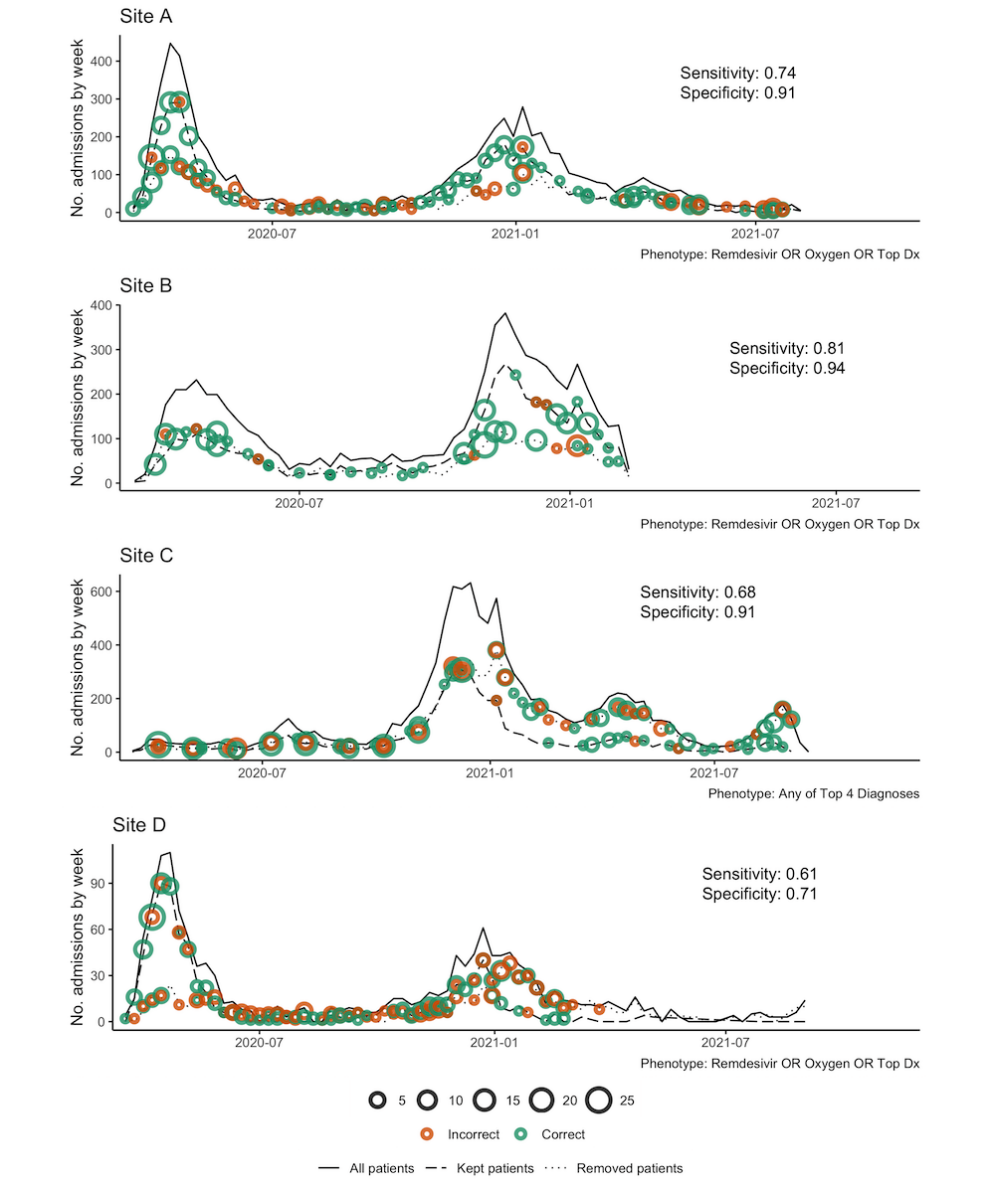
Performance of the top phenotyping feature sets (Table 7) over time at each site. The y axis is the number of admissions per week, the x axis is the week, and overall sensitivity and specificity are shown on each figure panel. Solid lines show the total number of weekly admissions for patients with a positive SARS-CoV-2 PCR test. Dashed lines show the number of weekly admissions after filtering to select patients admitted specifically for COVID-19 (ie, removing all patients who do not meet the phenotyping feature set criteria). The dotted line shows the difference between the solid line and the dashed line (ie, patients removed from the cohort in the dashed line). Green dots indicate correct classification by the phenotype according to chart review. Orange dots indicate incorrect classification. The dot size is proportional to the number of chart reviews. PCR: polymerase chain reaction.

We manually constructed 5 multisite phenotypes from elements in Table 7 that appeared at multiple sites. These were evaluated at each site: 2 variations of multisite diagnoses, 2 variations of all multisite features, and top laboratory tests. OR rules were favored due to better applicability across data sets (because of different coding practices at different sites), except for laboratory tests where the top pair of tests had high prevalence at every site. The best-performing phenotypes in each category are shown with their performance characteristics in Table 8, with the top single phenotype at each site in italics. In Figure 4, we plotted the performance of the top phenotype at each site (the boldfaced rows in Table 8) using the temporal phenotype visualization described in the Methods section. (The top phenotype involved all data types at every site except site C, where diagnoses alone performed better.)

**Table 8:** The best multisite phenotyping feature sets and their overall performance characteristics. The multisite phenotypes were derived from Table 7 by selecting components of phenotypes that appeared at multiple sites.

Phenotyping Feature Set	Description	Sensitivity, specificity
Other Viral Pneumonia OR Acute Respiratory Failure with Hypoxia OR Shortness of Breath OR Abnormal Lung Finding	*Retrospective phenotype:* Diagnoses mentioned in top feature sets at >1 site	Site A: 0.79,0.72 Site B: 0.88, 0.85 *Site C: 0.69,0.90^b^* Site D: 0.64,0.58
CRP^a^ AND Ferritin	*Real-time phenotype:* Laboratory tests mentioned in top feature sets at all 4 sites	Site A: 0.76,0.85 Site B: 0.88, 0.85 Site C: 0.42, 0.98 Site D: 0.66, 0.55
Remdesivir OR Oxygen (severe) OR Dx of Other Viral Pneumonia	*Retrospective phenotype:* All items mentioned at multiple sites in OR feature sets	*Site A: 0.74,0.91^b^* *Site B: 0.81, 0.94^b^* Site C: 0.60,0.92 *Site D: 0.61,0.71^b^*

^a^CRP: C-reactive protein.

^b^The top-performing phenotype at each site is italicized.

## Discussion

### Principal Results and Analysis

The COVID-19 pandemic has lasted for over 2 years, with multiple waves worldwide. Although hospital systems have been cyclically overwhelmed by patients seeking care for COVID-19, as health care systems began to open up before the second wave, elective surgeries were again performed starting in the later part of 2020, and especially in the second quarter of 2021, many approached the health care system for health issues (eg, accidents, strokes) while incidentally infected with SARS-CoV-2 [32]. This, along with the high false-positive rate of SARS-CoV-2 PCR tests in some situations [33-36], has led to increasing numbers of misclassified patients in analyses of COVID-19 characteristics and severity. This could be creating significant detection and reporting bias, leading to erroneous conclusions [10-13]. This study presents a multi-institutional characterization of 1123 hospitalized patients either incidentally infected with SARS-CoV-2 or specifically hospitalized for COVID-19 in 4 health care systems across multiple waves using consensus-based chart review criteria. Overall, we found that 764 (68%) of 1123 patients who tested SARS-CoV-2 positive were hospitalized because of COVID-19 but with significant variation during each wave of the pandemic.

We applied an item set–mining approach and established HSD principles to phenotype SARS-CoV-2 PCR-positive patients who were admitted specifically for COVID-19 by using data on charting patterns (eg, presence of laboratory tests within 48 hours of admission) rather than results (eg, laboratory results) [16,37]. HSD examines health care process data about a hospitalization, such as ordering/charting patterns, rather than the full data set. For example, to study severely ill patients, an HSD approach might select patients with a high total number of laboratory tests on the day of admission. This could be an indirect measure of clinical suspicion of disease complexity or severity. Previous work shows that proxies such as the total number of laboratory tests on the day of admission or the time of day of laboratory tests can be highly predictive of disease course [24,37]. Our methods sorted out who was treated for COVID-19 automatically, over time, with specificities above 0.70, even for some phenotypes discovered at a single site and applied to all 4. We focused on specificity because the goal was to remove false positives (ie, incidental SARS-CoV-2) from the cohort.

Our chart review protocol illustrates that patients who were admitted and had a positive SARS-CoV-2 PCR test were more likely to be admitted specifically for COVID-19 when disease prevalence was high (at least prior to Omicron). However, during periods in which health care systems were less restrictive (ie, resumed routine surgeries), a secondary measure/phenotype was critical for accurately classifying admissions specifically for SARS-CoV-2 infection.

As expected, we observed a lower proportion of hospitalizations specifically for COVID-19 in the summer months when disease prevalence was lower (Figure 3). One would expect this because there were fewer overall admissions as hospitals were recovering from the previous wave.

As expected, the top chart review criteria (Table 3) were *respiratory insufficiency* in admissions specifically for COVID-19 and *other* for incidental and uncertain admissions with SARS-CoV-2. Surprisingly, 10%-20% of patients admitted with incidental SARS-CoV-2 were diagnosed with pneumonia, respiratory failure, or acute kidney injury (Tables 5 and 6). This could reflect data collection issues, where some systems might repeat past problems automatically at hospital admission. In the case of codes for acute kidney injury, further investigation is needed to determine whether SARS-CoV-2-associated acute kidney injury (including COVID-19-associated nephropathy) occurs in patients we otherwise classified as having incidental admissions [38].

Health care systems are beginning to explore phenotyping feature sets to report admissions specifically for COVID-19. Starting January 2022 in Massachusetts, hospitals began reporting the number of for-COVID-19 hospitalizations as the count of admitted patients with both a SARS-CoV-2-positive test and a medication order for dexamethasone [22,23]. This simple phenotype was designed by the Massachusetts Department of Public Health as a first attempt, and it was based only on treatment recommendations for moderate-to-severe COVID-19 with hypoxia. It was not validated against a gold standard. Nonetheless, it illustrates the interest in EHR-based phenotyping for COVID-19.

Phenotypes with diagnosis codes tended to be the best-performing predictors of admissions, specifically for COVID-19. This could be because diagnosis codes represent either a clinically informed conclusion or a justification for ordering a test (implying the clinician suspected COVID-19). However, diagnoses are less prevalent in the population than laboratory tests and might not cover the entire population of admissions for COVID-19. Further, diagnoses early in hospitalization also do not always reflect the patient’s eventual diagnosis or hospital-related complications that are more accurately reflected in discharge diagnoses. There was also some heterogeneity in the diagnoses used at different sites (eg, B97.29 “Other Coronavirus as Cause of Disease” was a top predictor only at site B). In addition, the presence of laboratory tests is useful for real-time detection systems because diagnosis codes usually are assigned after discharge. Clusters of tests for inflammatory markers (eg, LDH, CRP, and ferritin) appeared across most sites as predictive of hospitalizations, specifically for COVID-19, which fits intuitively because an underlying systemic pathophysiological mechanism of SARS-CoV-2 is thought to be an inflammatory process [39,40], and guidelines therefore have encouraged health care providers to check inflammatory markers on COVID-19 admissions [41,42]. Many of these inflammatory laboratory tests are not routinely ordered on all hospitalized patients and would therefore be expected to help distinguish COVID-19 from other diseases. However, laboratory protocol differences across sites may have reduced generalizability for this metric.

Our methods generated pairs of items using OR and groups of up to 4 using AND logical operators. Our feature sets were somewhat vulnerable to the problem that specificity decreases when multiple elements are combined with OR, although, in general, OR feature sets performed better across sites because they could be designed to choose the top-performing elements at each site.

In addition to site differences, we also found changing disease management patterns over time. At the start of the pandemic, the only predictive phenotype was a pneumonia diagnosis. As standard COVID-19 order recommendations began to appear, laboratory orders became more consistent and predictive. Next, remdesivir began to be administered regularly. Finally, COVID-19-specific ICD-10 codes began to appear.

Overall, we found that an informatics-informed phenotyping approach successfully improved classification of for-COVID-19 versus incidental SARS-CoV-2-positive admissions, although generalizability was a challenge. Although some transfer learning is apparent (ie, a few phenotypes performed well across sites), local practice and charting patterns reduced generalizability. Specifically, phenotypes involving only laboratory tests did not perform well at site C, because the prevalence of these laboratory tests was low in the overall EHR data. This could be due to a data extraction or mapping issue in the underlying data warehouse. Site D had lower performance than other sites on the cross-site rules but not on the site-specific rules, perhaps highlighting less typical clinician treatment patterns.

Any of the multisite phenotypes developed here could be implemented as a cohort enhancement tool in hospital systems or data research networks, and the laboratory-only phenotypes (“CRP and Ferritin”) could be used for real-time corrections in reporting. However, because of the changing nature of COVID-19 and practice and coding variation across sites, these phenotypes should be used primarily as a starting point. It is important to run the phenotyping algorithm on each individual site’s data to tweak the rules to optimize them for each implementation.

### Limitations

Although the current data start at the beginning of the pandemic, they do not include the current Omicron wave nor much of the Delta wave. We believe that the techniques introduced here (if not the phenotypes themselves) will be applicable to these variants, and we are planning future studies to validate this.

Our phenotypes demonstrated some transfer learning but not enough to create a single phenotype applicable to all sites. Technically, our system used machine learning at individual sites, but results were manually aggregated across sites. Emerging techniques for federated learning [43] might reduce the manual work required and increase the complexity of possible cross-site phenotype testing.

Finally, an inherent weakness of EHR-based research is that EHR data do not directly represent the state of the patient, because some observations are not recorded in structured data and some entries in the EHR are made for nonclinical reasons (eg, to justify the cost of a test or to ensure adequate reimbursement for services). This is common to all EHR research efforts, and we mitigated this limitation by developing chart-verified phenotypes.

### Conclusion

At 4 health care systems around the United States over an 18-month period starting in March 2020, we developed and applied standardized chart review criteria to characterize the correct classification of hospitalization specifically for COVID-19 as compared to incidental hospitalization of a patient with a positive SARS-CoV-2 test or ICD-10 code. Then we applied HSD and frequent item set mining to electronic phenotyping to generate phenotypes specific to hospitalizations for COVID-19, and we showed how patterns changed over the course of the pandemic. Application of this approach could improve public health reporting, health care system resource disbursement, and research conclusions.

## Appendix

Multimedia Appendix 1 4CE data dictionary. 4CE: Consortium for Clinical Characterization of COVID-19 by EHR. EHR: electronic health record.

Multimedia Appendix 2 List of group members of the Consortium for Clinical Characterization of COVID-19 by EHR (4CE).

## Abbreviations

4CE: Consortium for Clinical Characterization of COVID-19 by EHR
BIDMC: Beth Israel Deaconess Medical Center
CRP: C-reactive protein
EHR: electronic health record
HSD: hospital system dynamics
ICD-10: International Classification of Diseases, Tenth Revision
IRB: Institutional Review Board
INR: international normalized ratio
LDH: lactate dehydrogenase
MGB: Mass General Brigham
NWU: Northwestern University
PCR: polymerase chain reaction
PPV: positive predictive value
UPITT: University of Pittsburgh/University of Pittsburgh Medical Center

## Data Availability

All data analysis code developed for this study is available under the Mozilla Public License v2 with a health care disclaimer.

The electronic health record (EHR) data sets analyzed during this study cannot be made publicly available due to regulations for protecting patient privacy and confidentiality. These regulations also prevent the data from being made available upon request from the authors. Any questions about the data set can be directed to the corresponding author.
