# Supplementary material for: Distinguishing Admissions Specifically for COVID-19 From Incidental SARS-CoV-2 Admissions: National Retrospective Electronic Health Record Study
Source: J Med Internet Res. 2022 May 18;24(5):e37931. doi: 10.2196/37931 (PMC9119395; doi:10.2196/37931)
Supplement: Multimedia Appendix 1 [file jmir_v24i5e37931_app1.pdf]

Appendix 1: 4CE EHR data

This is a list of all types of observations (facts about patients) that can appear in 4CE data sets. (Note that 4CE data sets are COVID-19-related.)

More information can be found online at https://github.com/covidclinical/PhaseX.2SqlDataExtraction

Diagnoses

Full ICD-9 or ICD-10 code

Procedures

Procedure Types
**Arterial Catheter**
**Bronchoscopy**
**CPR**
**Chest CT**
**ECMO**
**Emergency General Surgery**
**Emergency OBGYN**
**Emergency Orthopedics**
**Emergency Vascular Surgery**
**Renal Replacement**
**Supplemental Oxygen Other**
**Supplemental Oxygen Severe**

Table S1: Procedures that are defined by 4CE. Internally, these are built from a set of CPT codes in the raw EHR data.

Medications

4CE Code	***Definition***
**ACEI**	ACE inhibitors
**ARB**	Angiotensin II receptor blockers
**COAGA and COAGB**	Antithrombotic agents
**COVIDVIRAL**	COVID-related antivirals
**DIURETIC**	Diuretics
**HCQ**	Aminoquinolines
**ILI**	Interleukin inhibitor
**INTERFERON**	Interferon
**SIANES**	Anesthesia (indicating severe disease)
**SICARDIAC**	Cardiac-related (indicating severe disease)

Table S2: Medication categories defined by 4CE. Internally, these are built from a set of RxNorm codes in the raw EHR data.

Laboratory Tests

LOINC	*Lab Category*
**1742-6**	alanine aminotransferase (ALT)
**1751-7**	albumin
**1920-8**	aspartate aminotransferase (AST)
**1975-2**	total bilirubin
**1988-5**	C-reactive protein (CRP) (Normal Sensitivity)
**2019-8**	PaCO2
**2160-0**	creatinine
**2276-4**	Ferritin
**2532-0**	lactate dehydrogenase (LDH)
**2703-7**	PaO2
**3255-7**	Fibrinogen
**33959-8**	procalcitonin
**34714-6**	INR
**48065-7**	D-dimer (FEU)
**48066-5**	D-dimer (DDU)
**49563-0**	cardiac troponin (High Sensitivity)
**5902-2**	prothrombin time (PT)
**6598-7**	cardiac troponin (Normal Sensitivity)
**6690-2**	white blood cell count (Leukocytes)
**731-0**	lymphocyte count
**751-8**	neutrophil count
**777-3**	platelet count
**2019-8 / 2703-7**	Blood Gases
**UMLS C1335447**	COVID positive test
**UMLS C1334932**	COVID negative test

Table S3: Laboratory tests used by 4CE. Although most are assigned a LOINC code, the data might be mapped from multiple LOINC codes in the raw EHR data.
