# Supplementary material for: Distinguishing Admissions Specifically for COVID-19 From Incidental SARS-CoV-2 Admissions: National Retrospective Electronic Health Record Study
Source: J Med Internet Res. 2022 May 18;24(5):e37931. doi: 10.2196/37931 (PMC9119395; doi:10.2196/37931)
Supplement: Multimedia Appendix 2 [file jmir_v24i5e37931_app2.docx]

**List of group members of the Consortium for Clinical Characterization of COVID-19 by EHR (4CE).**

1. James R Aaron
2. Giuseppe Agapito
3. Adem Albayrak
4. Giuseppe Albi
5. Mario Alessiani
6. Anna Alloni
7. Danilo F Amendola
8. Li L.L.J Anthony
9. Bruce J Aronow
10. Fatima Ashraf
11. Andrew Atz
12. Paul Avillach
13. Paula S Azevedo
14. James Balshi
15. Brett K Beaulieu-Jones
16. Douglas S Bell
17. Antonio Bellasi
18. Riccardo Bellazzi
19. Vincent Benoit
20. Michele Beraghi
21. José Luis Bernal-Sobrino
22. Mélodie Bernaux
23. Romain Bey
24. Alvar Blanco-Martínez
25. Martin Boeker
26. Clara-Lea Bonzel
27. John Booth
28. Silvano Bosari
29. Florence T Bourgeois
30. Robert L Bradford
31. Gabriel A Brat
32. Stéphane Bréant
33. Carlos Tadeu Breda Neto
34. Nicholas W Brown
35. William A Bryant
36. Mauro Bucalo
37. Anita Burgun
38. Tianxi Cai
39. Mario Cannataro
40. Aldo Carmona
41. Charlotte Caucheteux
42. Julien Champ
43. Jin Chen
44. Krista Chen
45. Luca Chiovato
46. Lorenzo Chiudinelli
47. Kelly Cho
48. James J Cimino
49. Tiago K Colicchio
50. Sylvie Cormont
51. Sébastien Cossin
52. Jean B Craig
53. Juan Luis Cruz-Bermúdez
54. Jaime Cruz-Rojo
55. Arianna Dagliati
56. Mohamad Daniar
57. Christel Daniel
58. Priyam Das
59. Batsal Devkota
60. Julien Dubiel
61. Loic Esteve
62. Hossein Estiri
63. Shirley Fan
64. Robert W Follett
65. Thomas Ganslandt
66. Noelia García-Barrio
67. Lana X Garmire
68. Nils Gehlenborg
69. Emily Getzen
70. Alon Geva
71. Tobias Gradinger
72. Alexandre Gramfort
73. Romain Griffier
74. Nicolas Griffon
75. Olivier Grisel
76. Alba Gutiérrez-Sacristán
77. Larry Han
78. David A Hanauer
79. Christian Haverkamp
80. Bing He
81. Darren W Henderson
82. Martin Hilka
83. Yuk-Lam Ho
84. John H Holmes
85. Chuan Hong
86. Kenneth M Huling
87. Meghan R Hutch
88. Richard W Issitt
89. Anne Sophie Jannot
90. Vianney Jouhet
91. Mark S Keller
92. Chris J Kennedy
93. Daniel A Key
94. Katie Kirchoff
95. Jeffrey G Klann
96. Isaac S Kohane
97. Ian D Krantz
98. Detlef Kraska
99. Ashok K Krishnamurthy
100. Sehi L'Yi
101. Trang T Le
102. Judith Leblanc
103. Guillaume Lemaitre
104. Leslie Lenert
105. Damien Leprovost
106. Molei Liu
107. Ne Hooi Will Loh
108. Qi Long
109. Sara Lozano-Zahonero
110. Yuan Luo
111. Kristine E Lynch
112. Sadiqa Mahmood
113. Sarah Maidlow
114. Adeline Makoudjou
115. Alberto Malovini
116. Kenneth D Mandl
117. Chengsheng Mao
118. Anupama Maram
119. Patricia Martel
120. Marcelo R Martins
121. Jayson S Marwaha
122. Aaron J Masino
123. Maria Mazzitelli
124. Arthur Mensch
125. Marianna Milano
126. Marcos F Minicucci
127. Bertrand Moal
128. Taha Mohseni Ahooyi
129. Jason H Moore
130. Cinta Moraleda
131. Jeffrey S Morris
132. Michele Morris
133. Karyn L Moshal
134. Sajad Mousavi
135. Danielle L Mowery
136. Douglas A Murad
137. Shawn N Murphy
138. Thomas P Naughton
139. Antoine Neuraz
140. Kee Yuan Ngiam
141. Wanjiku FM Njoroge
142. James B Norman
143. Jihad Obeid
144. Marina P Okoshi
145. Karen L Olson
146. Gilbert S Omenn
147. Nina Orlova
148. Brian D Ostasiewski
149. Nathan P Palmer
150. Nicolas Paris
151. Lav P Patel
152. Miguel Pedrera-Jimenez
153. Emily R Pfaff
154. Ashley C Pfaff
155. Danielle Pillion
156. Sara Pizzimenti
157. Hans U Prokosch
158. Robson A Prudente
159. Andrea Prunotto
160. Víctor Quirós-González
161. Rachel B Ramoni
162. Maryna Raskin
163. Siegbert Rieg
164. Gustavo Roig-Domínguez
165. Pablo Rojo
166. Paula Rubio-Mayo
167. Carlos Sáez
168. Elisa Salamanca
169. Malarkodi J Samayamuthu
170. L. Nelson Sanchez-Pinto
171. Arnaud Sandrin
172. Nandhini Santhanam
173. Janaina CC Santos
174. Fernando J Sanz Vidorreta
175. Maria Savino
176. Emily R Schriver
177. Petra Schubert
178. Juergen Schuettler
179. Luigia Scudeller
180. Neil J Sebire
181. Pablo Serrano-Balazote
182. Patricia Serre
183. Arnaud Serret-Larmande
184. Mohsin Shah
185. Zahra Shakeri
186. Domenick Silvio
187. Piotr Sliz
188. Jiyeon Son
189. Charles Sonday
190. Andrew M South
191. Anastasia Spiridou
192. Zachary H Strasser
193. Amelia LM Tan
194. Bryce WQ Tan
195. Byorn WL Tan
196. Suzana E Tanni
197. Deanne M Taylor
198. Ana I Terriza-Torres
199. Valentina Tibollo
200. Patric Tippmann
201. Emma MS Toh
202. Carlo Torti
203. Enrico M Trecarichi
204. Yi-Ju Tseng
205. Andrew K Vallejos
206. Gael Varoquaux
207. Margaret E Vella
208. Guillaume Verdy
209. Jill-Jênn Vie
210. Shyam Visweswaran
211. Michele Vitacca
212. Kavishwar B Wagholikar
213. Lemuel R Waitman
214. Xuan Wang
215. Demian Wassermann
216. Griffin M Weber
217. Martin Wolkewitz
218. Scott Wong
219. Zongqi Xia
220. Xin Xiong
221. Ye Ye
222. Nadir Yehya
223. William Yuan
224. Alberto Zambelli
225. Harrison G Zhang
226. Daniel Zoeller
227. Chiara Zucco
